# Melatonin as an adjuvant therapy in gestational hypertension and pre-eclampsia: A randomized controlled trial

**DOI:** 10.6026/973206300214479

**Published:** 2025-12-15

**Authors:** Akanksha Lamba Thora, Pankhudi Singh Jyoti, Anupama Dave

**Affiliations:** 1 Department of Obstetrics and Gynaecology, MTH Hospital, Indore Madhya Pradesh, India; 2 Department of Obstetrics and Gynaecology, MTH Women's Hospital, MGM Medical College, Indore, Madhya Pradesh, India

**Keywords:** Melatonin, pregnancy, hypertension, pre-eclampsia, adjuvant therapy

## Abstract

In spite of the improvement of obstetric care, hypertensive disorders of pregnancy have continued to emerge as the major causes of maternal and perinatal morbidity. Therefore, it is of interest to assess the effectiveness of melatonin as an adjuvant treatment in the management of gestational hypertension and pre-eclampsia in 404 pregnant women (183 taking melatonin and 221 taking standard care) in one of the tertiary care hospitals in India. Melatonin group also showed much lower incidences of serious pre-eclampsia that necessitated cesarean section (2.73% vs 8.60, p=0.014), lower rate of proteinuria (28.42 vs 37.10), and a better fetal outcome with a lower incidence of intrauterine death (4.92 vs 9.50). Melatonin supplementation is a potential adjuvant treatment that is promising to decrease the maternal and infant complications in pregnancy-related hypertension disorders.

## Background:

Hypertensive pregnancy disorders, which include gestational hypertension, pre-eclampsia, eclampsia, and HELLP syndrome, occur in about 5-10 percent of all pregnancies across the world and are the major cause of maternal and perinatal deaths [1]. Pre-eclampsia in itself causes problems in 2-8 percent of pregnancies worldwide and even greater rates of 5-15 percent have been reported in developing nations such as India [2]. This is a disease that presents with new-onset hypertension preceded by 20 weeks of gestation, which is usually accompanied by proteinuria and dysfunction of end-organisms [3]. Although there is an increase in prenatal surveillance and timely birth, the disorders have persisted as the leading causes of maternal morbidity that includes cerebrovascular accidents, renal failure, hepatic dysfunction and coagulopathy [4]. Pathophysiology Pre-eclampsia is associated with impaired placentation, endothelial dysfunction, oxidative stress, and systemic inflammation [5]. The current management techniques are mainly aimed at the management of blood pressure using antihypertensive drugs, prophylaxis of seizures using magnesium sulfate, timely delivery [6]. These however treat symptoms and not pathophysiological mechanisms, which definitive treatment is delivered, which in most cases needs preterm birth and neonatal complications [7]. The neurohormone that has been developed as a possible treatment option is melatonin, which is secreted by the pineal glands and the placenta against the background of its antioxidant, anti-inflammatory, and vasodilatory effects [8]. Melatonin passes freely through the placenta and is involved in various important functions of circadian regulation, free radicals scavenging, and immunological regulation [9]. Recent data indicate that women with pre-eclampsia have lower levels of the hormone melatonin, and supplementation can be used to enhance the endothelial activity and minimize oxidative stress [10]. An initial study by Hobson *et al.* proved that the use of melatonin in early pre-eclampsia increased the duration of pregnancy by around six days, and lowered the necessity to increase antihypertensive treatment [11]. Nevertheless, there are scanty large-scale randomized controlled trials on the effectiveness of melatonin in gestational hypertension and pre-eclampsia especially in the South Asian population where the disease affliction is high. Therefore, it is of interest to determine the role of melatonin as an adjuvant medication in the treatment of gestational hypertension and pre-eclampsia with particular attention to the maternal and fetal outcomes.

## Materials and Methods:

## Study design and setting:

It was a prospective, randomized controlled interventional trial carried out at MTH Hospital, Indore, Madhya Pradesh, India, in a 12-month period between January 2024 and December 2024 after the institutional ethics committee gave its approval.

## Population and sample size of the study:

The eligibility was screened by pregnant women who came to the hospital with gestational age more than 28 weeks and continued with high blood pressure (≥140/90 mmHg at least twice, four hours separate). The calculated sample of the study was as follows: n=130 /group, Z=0.96 (95% confidence level), P=0.25 (probability of expected prevalence), Q=1.00 (probability of 75 percent), d=0.075 (margin of error), which is 130 participants in every group. Taking into consideration the possibility of loss to follow-up, we were planning to recruit 250 participants on each group. The last research group included 404 women, 183 in intervention (cases) and 221 in control group.

## Inclusion and exclusion criteria:

The inclusion criteria included: pregnant women who were aged above 18 years, had a gestational age above 28 weeks and had continued hypertension (BP ≥140/90 mmHg) and gave written informed consent. The exclusion criteria were: The patient had chronic hypertension before pregnancy, presented with eclampsia, taking sedative drugs and lack of willingness to join.

## Randomization and intervention:

The participants who were eligible were randomly assigned using an odd-even allocation criterion depending on the number of their hospital registration. Females in the intervention group were given 30 mg of oral melatonin at bedtime daily in addition to regular antihypertensive medication whereas the control group was given the regular care. The standard management comprised of antihypertensive drugs (methyldopa, labetalol, or nifedipine as indicated), magnesium sulfate in case of severe pre-eclampsia and timely birth. The administration of melatonin was until childbirth.

## Data collection and Following-up:

Baseline information was documented in detail such as age, parity, socioeconomic status, gestational age at enrolment and medical history. The subjects had a biweekly follow up and their blood pressure (measured in sitting position using standard mercury sphygmomanometer), level of urine albumin (monitored by using color coded dipstick strip) and clinical symptoms were observed. As clinically indicated, laboratory tests, such as complete blood count, liver functioning tests, renal functioning tests, and coagulation profile, were conducted. Funds examination was also done to identify hypertensive retinopathy.

## Outcome measures:

Main ones were: maternal complications (progression to severe pre-eclampsia, eclampsia, proteinuria, thrombocytopenia, deranged liver/renal function, HELLP syndrome, abnormal fundus findings) and mode of delivery. Second outcomes were: gestational age at birth, fetal outcomes (intrauterine death, special newborn care unit/high-dependency unit admission, healthy baby), and cesarean section indications.

## Statistical analysis:

The data were tabulated using the Microsoft Excel 2019 and were processed using SPSS version 21.0 (IBM, Armonk, NY, USA). Frequencies and percentages were used to describe categorical variables and compared between them with Chi-square test or Fisher's exact test based on the case. Mean standard deviation was used to show continuous variables and to compare them with independent t-test. The statistical significance of all the analyses was taken to be p-value below 0.05.

## Results:

A total of 404 pregnant women with gestational hypertension or pre-eclampsia were enrolled and analyzed (183 in the melatonin group and 221 in the control group). The baseline demographic and clinical characteristics were comparable between groups (Table 1 - see PDF). The majority of participants were aged 18-25 years (48.63% in cases vs 44.80% in controls), with no significant difference in age distribution (p=0.055). Socioeconomic status and parity were similarly distributed between groups (p=0.292 and p=0.434, respectively). However, gestational age at enrollment differed significantly, with more women in the case group enrolled between 32-36 weeks (55.19% vs 37.56%, p<0.001). Maternal complications were evaluated comprehensively (Table 2 - see PDF). The incidence of proteinuria (urine albumin ≥+1) was lower in the melatonin group compared to controls (28.42% vs 37.10%), approaching statistical significance (p=0.064). Eclampsia occurred in 6.01% of the melatonin group versus 6.79% of controls (p=0.751). Thrombocytopenia was observed in 12.02% of cases and 8.60% of controls (p=0.250). Deranged liver or renal function tests occurred in 4.92% versus 6.33% (p=0.540), while HELLP syndrome affected 1.64% versus 2.26% (p=0.654) of participants in the melatonin and control groups, respectively. Regarding delivery outcomes, preterm delivery rates were similar between groups (50.27% vs 48.87%, p=0.778). The mode of delivery showed a non-significant trend toward more vaginal deliveries in the melatonin group (65.03% vs 57.47%, p=0.121). Among indications for cesarean section, severe pre-eclampsia was significantly less common in the melatonin group (2.73% vs 8.60%, p=0.014), while other indications including failed induction, meconium-stained liquor with fetal distress, and previous cesarean sections showed no significant differences. Fetal outcomes demonstrated favorable trends in the melatonin group (Table 3 - see PDF). The incidence of intrauterine fetal death was lower in the melatonin group (4.92% vs 9.50%), although this did not reach statistical significance (p=0.058). The proportion of healthy babies without requiring neonatal intensive care was higher in the melatonin group (50.82% vs 45.70%). Admission rates to special newborn care unit or high-dependency unit were comparable between groups (44.26% vs 44.80%).

## Discussion:

This randomized controlled trial compared the use of melatonin as an adjuvant intervention in gestational hypertension and pre-eclampsia and has provided a number of clinically relevant results. The women who were given melatonin supplementation reported a significant decrease of progression to severe pre-eclampsia that required cesarean delivery, decreased proteinuria, and better fetal outcomes in comparison to the conventional care. The clinical value of the much lower incidence of severe pre-eclampsia as a cesarean section indicator in the melatonin group (2.73-8.60, p=0.014) is a key clinical advantage. This observation is similar to the pilot study by Hobson *et al.* that stated that when melatonin was administered in early pre-eclampsia, it extended the pregnancy and decreased the necessity to increase the dose of antihypertensive treatment [11]. The pathophysiological mechanism working on this benefit could be the strong antioxidant effect of melatonin and the capability to reduce endothelial dysfunction, which is a core pathophysiology of pre-eclampsia [12]. Melatonin removes reactive oxygen species and increases antioxidant enzymes and hence it lowers oxidative alleviation induced placental and vascular harm [13]. A negative trend in proteinuria, which was observed in the melatonin group (28.42% vs 37.10, p=0.064) indicates renal protection. The proteinuria associated with pre-eclampsia is caused by endotheliosis of glomeruli and disappearance of glomerular barrier integrity [14]. These pathological changes can be countered with the anti-inflammatory and endothelial-protective effects of melatonin. The findings of animal experiments proved that melatonin decreases proteinuria and maintains renal activity in models of hypertensive pregnancy by regulating the renin-angiotensin system and suppressing the production of inflammatory cytokines [15].

This higher rate of intrauterine fetal death in the melatonin group (4.92 vs 9.50), though not significant statistically, is a trend of clinical significance. Pre-eclampsia impairs the uteroplacental perfusion and results in fetal growth retardation and high mortality chances [16]. The vasodilatory effect and capacity of melatonin to increase placental performance could result in improved fetal ventilation and nutrient supply [17]. Moreover, melatonin diffuses through the placenta and offers direct antioxidant defense to the unborn baby and may decrease hypoxic-ischemic damage [18]. The similar incidences of other complications associated with maternal conditions in the two groups indicate that melatonin was not influential in the overall course of disease in already existing hypertensive disorders. This observation is in line with the existing knowledge that melatonin could be most effective when administered at an early stage of pregnancy or prior to the appearance of pronounced symptoms of the disease [19]. The future research should examine appropriate times and periods of melatonin supplementation in order to reap maximum therapeutic effects. The strengths of our study are a random design, relatively high sample size, and the evaluation of maternal and fetal outcomes. Nevertheless, such limitations are the open-label design, single-centered environment, and the lack of stratification of the findings according to the severity of the disease at the baseline. Also, loss to follow-up was more in melatonin group (26.8% vs 11.6) and this can be a source of bias. Biomarker analysis did not investigate the mechanisms of the effects of melatonin, which restricted the mechanistic understanding. Evidence available regarding the safety of melatonin use during pregnancy is positive [20]. No severe side effects that can be associated with melatonin were identified in our research, which was in line with the previous findings. Melatonin is a naturally occurring hormone produced in pregnancy and has been safely administered in a number of clinical situations. Nevertheless, the long-term developmental process of offspring needs to be researched with the extended follow-up.

## Conclusion:

Judicious use of melatonin supplementation as an adjuvant treatment in gestational hypertension and pre-eclampsia had a significant negative outcome by decreasing the severity of pre-eclampsia that leads to operation and demonstrated tendencies toward better renal protection and better birth outcomes. These results justify the possible use of melatonin as a safe and inexpensive supplementary component of the routine management studies in resource-deprived conditions.

## References

[1] https://pubmed.ncbi.nlm.nih.gov/32443079/.

[2] Magee L.A (2019). PLoS Med..

[3] Rana S (2019). Circ Res..

[4] Mol B.W.J (2016). Lancet..

[5] Phipps E.A (2019). Nat Rev Nephrol..

[6] Abalos E (2018). Cochrane Database Syst Rev..

[7] Chappell L.C (2019). Lancet..

[8] Reiter R.J (2014). Hum Reprod Update..

[9] Lanoix D (2012). J Pineal Res..

[10] Bouchlariotou S (2014). Ren Fail..

[11] Hobson S.R (2018). J Pineal Res..

[12] Langston-Cox A (2021). Antioxidants (Basel)..

[13] Reiter R.J (2017). Int J Mol Sci..

[14] Garovic V.D (2007). Nephrol Dial Transplant..

[15] Zuo J, Jiang Z (2020). Vasc Med..

[16] Lisonkova S, Joseph K.S (2013). Am J Obstet Gynecol..

[17] Sagrillo-Fagundes L (2018). J Pineal Res..

[18] Miller S.L (2014). J Pineal Res..

[19] Fantasia I (2022). Eur J Obstet Gynecol Reprod Biol..

[20] Verteramo R (2022). Biomedicines..

